# Rational Prescribing in Primary Care (RaPP): A Cluster Randomized Trial of a Tailored Intervention

**DOI:** 10.1371/journal.pmed.0030134

**Published:** 2006-06-06

**Authors:** Atle Fretheim, Andrew D Oxman, Kari Håvelsrud, Shaun Treweek, Doris T Kristoffersen, Arild Bjørndal

**Affiliations:** **1**Norwegian Knowledge Centre for Health Services, Oslo, Norway; **2**Tayside Centre for General Practice, University of Dundee, Dundee, United Kingdom; George Institute for International HealthUnited States of America

## Abstract

**Background:**

A gap exists between evidence and practice regarding the management of cardiovascular risk factors. This gap could be narrowed if systematically developed clinical practice guidelines were effectively implemented in clinical practice. We evaluated the effects of a tailored intervention to support the implementation of systematically developed guidelines for the use of antihypertensive and cholesterol-lowering drugs for the primary prevention of cardiovascular disease.

**Methods and Findings:**

We conducted a cluster-randomized trial comparing a tailored intervention to passive dissemination of guidelines in 146 general practices in two geographical areas in Norway. Each practice was randomized to either the tailored intervention (70 practices; 257 physicians) or control group (69 practices; 244 physicians). Patients started on medication for hypertension or hypercholesterolemia during the study period and all patients already on treatment that consulted their physician during the trial were included. A multifaceted intervention was tailored to address identified barriers to change. Key components were an educational outreach visit with audit and feedback, and computerized reminders linked to the medical record system. Pharmacists conducted the visits. Outcomes were measured for all eligible patients seen in the participating practices during 1 y before and after the intervention. The main outcomes were the proportions of (1) first-time prescriptions for hypertension where thiazides were prescribed, (2) patients assessed for cardiovascular risk before prescribing antihypertensive or cholesterol-lowering drugs, and (3) patients treated for hypertension or hypercholesterolemia for 3 mo or more who had achieved recommended treatment goals.

The intervention led to an increase in adherence to guideline recommendations on choice of antihypertensive drug. Thiazides were prescribed to 17% of patients in the intervention group versus 11% in the control group (relative risk 1.94; 95% confidence interval 1.49–2.49, adjusted for baseline differences and clustering effect). Little or no differences were found for risk assessment prior to prescribing and for achievement of treatment goals.

**Conclusions:**

Our tailored intervention had a significant impact on prescribing of antihypertensive drugs, but was ineffective in improving the quality of other aspects of managing hypertension and hypercholesterolemia in primary care.

## Editors' Summary

**Background.:**

An important issue in health care is “getting research into practice,” in other words, making sure that, when evidence from research has established the best way to treat a disease, doctors actually use that approach with their patients. In reality, there is often a gap between evidence and practice.

An example concerns the treatment of people who have high blood pressure (hypertension) and/or high cholesterol. These are common conditions, and both increase the risk of having a heart attack or a stroke. Research has shown that the risks can be lowered if patients with these conditions are given drugs that lower blood pressure (antihypertensives) and drugs that lower cholesterol. There are many types of these drugs now available. In many countries, the health authorities want family doctors (general practitioners) to make better use of these drugs. They want doctors to prescribe them to everyone who would benefit, using the type of drugs found to be most effective. When there is a choice of drugs that are equally effective, they want doctors to use the cheapest type. (In the case of antihypertensives, an older type, known as thiazides, is very effective and also very cheap, but many doctors prefer to give their patients newer, more expensive alternatives.) Health authorities have issued guidelines to doctors that address these issues. However, it is not easy to change prescribing practices, and research in several countries has shown that issuing guidelines has only limited effects.

**Why Was This Study Done?:**

The researchers wanted—in two parts of Norway—to compare the effects on prescribing practices of what they called the “passive dissemination of guidelines” with a more active approach, where the use of the guidelines was strongly promoted and encouraged.

**What Did the Researchers Do and Find?:**

They worked with 146 general practices. In half of them the guidelines were actively promoted. The remaining were regarded as a control group; they were given the guidelines but no special efforts were made to encourage their use. It was decided at random which practices would be in which group; this approach is called a randomized controlled trial. The methods used to actively promote use of the guidelines included personal visits to the practices by pharmacists and use of a computerized reminder system. Information was then collected on the number of patients who, when first treated for hypertension, were prescribed a thiazide. Other information collected included whether patients had been properly assessed for their level of risk (for strokes and heart attacks) before antihypertensive or cholesterol-lowering drugs were given. In addition, the researchers recorded whether the recommended targets for improvement in blood pressure and cholesterol level had been reached.

Only 11% of those patients visiting the control group of practices who should have been prescribed thiazides, according to the guidelines, actually received them. Of those seen by doctors in the practices where the guidelines were actively promoted, 17% received thiazides. According to statistical analysis, the increase achieved by active promotion is significant. Little or no differences were found for risk assessment prior to prescribing and for achievement of treatment goals.

****What Do These Findings Mean?**:**

Even in the active promotion group, the great majority of patients (83%) were still not receiving treatment according to the guidelines. However, active promotion of guidelines is more effective than simply issuing the guidelines by themselves. The study also demonstrates that it is very hard to change prescribing practices. The efforts made here to encourage the doctors to change were considerable, and although the results were significant, they were still disappointing. Also disappointing is the fact that achievement of treatment goals was no better in the active-promotion group. These issues are discussed further in a Perspective about this study (DOI: 10.1371/journal.pmed.0030229).

**Additional Information.:**

Please access these Web sites via the online version of this summary at http://dx.doi.org/10.1371/journal.pmed.0030134.

• The Web site of the American Academy of Family Physicians has a page on heart disease

• The MedlinePlus Medical Encyclopedia's pages on heart diseases and vascular diseases

• Information from NHS Direct (UK National Health Service) about heart attack and stroke

• Another PLoS Medicine article has also addressed trends in thiazide prescribing

## Abstract 3

Passive dissemination of management guidelines for hypertension and hypercholesterolaemia was compared with active promotion. Active promotion led to significant improvement in antihypertensive prescribing but not other aspects of management.

## Introduction

Hypertension and hypercholesterolemia are common problems in general practice and have important consequences for patients and the use of health-care resources.

There is high-quality evidence from well-designed randomized trials of the effects of antihypertensive and cholesterol-lowering drugs for the primary prevention of cardiovascular disease [1,2]. However, a gap exists between evidence and practice regarding several aspects of cardiovascular risk factor management [3]. This gap could be narrowed if systematically developed clinical practice guidelines were effectively implemented. Several randomized trials of guideline implementation strategies have been conducted, generally demonstrating only modest effects [4].

Based on a review of previous guidelines and the underlying evidence, we developed guidelines for prescribing antihypertensive and cholesterol-lowering drugs for the primary prevention of cardiovascular disease in Norwegian general practice [5–7].

We decided to address possible improvements for three key issues: cardiovascular risk is commonly not estimated before starting treatment [8], thiazides are underutilized when initiating treatment for hypertension [9], and treatment goals are frequently not achieved for both blood pressure and cholesterol [10,11]. We therefore developed an intervention aimed at improving performance in these three areas and conducted a trial to assess the effectiveness and costs of this intervention.

The results of evaluations of interventions to improve clinical practice differ, and large effects are not common [12,13]. As with any behavior, professional behavior is difficult to change. The underlying reasons for differences between clinical practice and recommendations in systematically developed guidelines vary from one clinical problem to another and from one clinician to another [14,15]. It is therefore logical to attempt to tailor strategies to support the implementation of guidelines to address identified barriers to change [15]. However, the effectiveness of tailored interventions is uncertain [16].

The primary objective of this trial was to evaluate the effects of a tailored intervention to support the implementation of guidelines in Norwegian general practice for the use of antihypertensive and cholesterol-lowering drugs for the primary prevention of cardiovascular disease. Our hypothesis was that the tailored intervention would be more effective than passive dissemination of the guidelines.

## Methods

We anticipated that passive dissemination of guidelines would have little effect on the outcomes of interest [12,13,18] and therefore was an appropriate control intervention. We decided that randomizing practices rather than physicians or patients would be the most feasible design for practical reasons, and also the most appropriate to avoid contamination from the intervention to the control group.

The methods have been described in more detail in the study protocol (Protocol S1; [18]).

### Participants

The study participants were all general practices in two geographically defined areas of Norway; the practices all used one or the other of two eligible electronic medical record systems.

Patients started on medication for hypertension or hypercholesterolemia during the study period and all patients already on treatment that consulted their physician during the trial were included in the analyses. The eligibility criteria for patients are described in more detail under “Outcomes.”

### Interventions

We developed our intervention through a process of identifying barriers to implementation of the recommendations and tailoring the intervention to address these. This is described in more detail elsewhere [19]. Box 1 shows the various elements of our intervention.

The intervention was initiated through an educational outreach visit carried out between May and December 2002 by pharmacists recruited and trained specifically for this purpose.

During the outreach visit the main elements of the guidelines were presented, with special emphasis on cardiovascular risk estimation, choice of first-line drugs for hypertension, and treatment goals. A printed copy of the guidelines and a one-page version were given to the physicians, including a chart to aid the estimation of cardiovascular risk.

A software package was installed during the visit. This enabled us to extract data and immediately, during the visit, present the physicians with data on their performance of risk estimation, choice of antihypertensive drugs, and achievement of treatment goals (audit and feedback). The software package also included computerized reminders (“pop-ups”) on the computer screen. These were triggered at the patient's first visit following a recording of an elevated blood pressure (>140/90 mm Hg) or cholesterol level (total cholesterol > 5 mmol/l [190 mg/dl] or low-density lipoprotein [LDL] cholesterol > 3 mmol/l [115 mg/dl]).

If the patient had not been prescribed blood-pressure- or cholesterol-lowering drugs, the physician was reminded of the recommendation to carry out cardiovascular risk assessment and was given the option of starting a computer program for risk assessment. Recommendations on choice of drugs were also given, and the physician was given the choice of printing out patient information material.

If the patient was already on blood-pressure- or cholesterol-lowering drugs, the pop-up reminded the physician of recommended treatment goals and asked if the physician would like to print out patient information material.

Within 3 d after the outreach visit, a member of the research team called the clinic to confirm that they were not experiencing problems with their computers as a result of our visit.

The doctors who were invited to participate in the study were given information about the objectives of the study and the practical impact it might have on their practice. We obtained written consent from all practices. We submitted the research protocol to the Regional Committee of Research Ethics, which considered ethical approval unnecessary.

The Norwegian Data Inspectorate approved the handling of the data.

### Outcomes

We chose three main outcome measures, all aimed at physician behavior regarding the pharmacological management of primary prevention of cardiovascular disease for the 12 mo following the outreach visit. All outcomes were recorded at the patient level. Because the eligibility criteria varied from one outcome to another, the number of patients included in the analysis varied across outcomes. Baseline data for the 12 mo preceding the intervention were also collected.

Patients with established cardiovascular disease were excluded, with the exception of the outcomes related to treatment goals for lipid-lowering therapy, since these are the same across patient groups. Because antihypertensive drugs are also prescribed for the treatment of thyrotoxicosis and migraine, we excluded data from patients with these diagnoses. We used extracted medical record data on prescribing and diagnoses to identify patients who were to be excluded. For example, patients who had been given a prescription for nitroglycerin were assumed to have coronary heart disease, and thus were excluded from most analyses.

Patients were considered to be previously untreated if they had hypertension (blood pressure > 140/90 mm Hg) or hypercholesterolemia (total cholesterol > 5 mmol/l [190 mg/dl] or LDL cholesterol > 3 mmol/l [115 mg/dl]) but no prescription for the corresponding medication had been recorded for 24 mo preceding the outreach visit.

The primary outcomes were the following: (1) the proportion of patients prescribed thiazides among patients prescribed antihypertensive drugs for the first time, (2) the proportion of patients with a cardiovascular risk assessment among all those started on antihypertensive or cholesterol-lowering treatment (excluding patients already on either type of medication), and (3) the proportion of patients with a recorded level of cholesterol (total or LDL) or blood pressure satisfying the specified treatment goals among all patients on the corresponding treatment for at least 3 mo. For cholesterol we also included patients on secondary prevention since the treatment goals are similar.

The secondary outcomes, as prespecified in the research protocol (Protocol S1; [18]), were the following: (1) the proportion of patients reporting that they were involved in the decision-making process before drug treatment for hypertension and/or elevated cholesterol was started, (2) the level of risk among patients started on treatment, (3) the proportion of patients with risk above 20% among those started on treatment, (4) the level of risk among patients not started on treatment for whom blood pressure and cholesterol level were recorded, (5) the proportion of prescriptions of thiazides or beta-blockers to patients who were prescribed antihypertensive drugs for the first time, (6) the proportion of prescriptions of angiotensin receptor blockers or alpha-blockers to patients who were prescribed antihypertensive drugs for the first time, (7) for patients with diabetes, the proportion of patients with a recorded level of cholesterol (total or LDL) or hypertension satisfying the specified treatment goals among all patients on the corresponding treatment (for cholesterol we also included patients on secondary prevention since the treatment goals are similar), (8) the proportion of patients reaching the specified treatment goal for blood pressure, and (9) the proportion of patients reaching the specified treatment goal for cholesterol level.

All outcomes were calculated from data extracted from the practices' medical record systems, with two exceptions: (1) the use of cardiovascular risk assessment tools by physicians before starting medication and (2) the level of patient involvement. Patients potentially eligible for inclusion in these analyses were identified from the medical record data, after which we interviewed the prescribing physician (or a colleague) per telephone. We enquired about the use of risk assessment tools for each patient they had started on treatment during the intervention period, and the physicians gave their answers based on notes from the medical records. The physicians assisted us by sending a questionnaire to the patients who had been started on medication. The questionnaire consisted of one question, asking to what extent the patients felt that they had taken part in the decision to start drug therapy. The answer was given on a five-point scale, from “none” to “fully.” Only patients reporting that they took no part at all were counted as not being involved in the decision to start treatment.

For two outcomes—(1) the proportion of patients for whom cardiovascular risk had not been estimated and (2) the level of risk among patients not started on treatment—the analysis was based on a random sample of eligible patients.

During interviews with physicians it was often possible to figure out whether a practice was in the intervention group. Investigators assessing outcomes and conducting analyses were blinded to the allocation of practices.

All analyses were by intention to treat. In response to comments from peer reviewers, we decided to deviate from our protocol and use adherence, rather than non-adherence, to recommendations as outcome measures. This had no impact on our findings.

### Sample Size

In order to demonstrate a 25% relative reduction in non-adherence with the guidelines—with a power of 80% and a statistical significance level of 5%—in outcome measures between the control and intervention groups, we estimated that we needed a sample of approximately 140 practices in total (Cluster Randomisation Sample Size Calculator version 1.0.2, Health Services Research Unit, University of Aberdeen, Aberdeen, United Kingdom). The adjusting factor (intracluster correlation coefficient) was conservatively estimated to be 0.2, based on data from a previous study [20]. More detail is available elsewhere [18].

### Randomization

Block randomization was done within two geographical areas (Oslo and Tromsø) to ensure balance in the number of practices in the intervention and control groups. The size of the blocks varied randomly between two, four, and six. A colleague not directly involved in our research project generated the allocation list using software from http://www.randomization.com. We gave her identification numbers representing each recruited practice, and she informed us whether the practice was allocated to the intervention or control group.

### Statistical Methods

The generalized estimating equations (GEE) approach was used for the analysis of the binary outcomes, and the mixed effects linear regression model (a two-stage nested analysis of variance) for the continuous outcome (risk level), using the baseline log odds for compliance and the baseline mean risk level for each practice, respectively, as covariates [21]. The analysis was performed using PROC GENMOD and PROC MIXED in SAS 9.1.3 (SAS Institute, Cary, North Carolina, United States).

The outcome from a GEE approach is an odds ratio; the adjusted relative risk was calculated from the odds ratio by using the overall proportion of compliance in the control group [22].

Originally we planned to use a parametric method, which depends on an underlying distribution of the sample observations. This is not the case for the GEE approach. We therefore used the GEE approach rather than the method we had specified in the protocol. This change was made before we analyzed the data.

## Results

### Participant Flow

**Figure 1:**
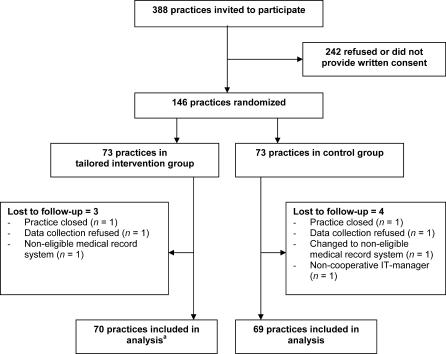
Flow of Practices through Trial ^a^For two of these practices no outreach visit was conducted (one withdrew after randomization and one had technical problems) but data collection was possible.

Figure 1 illustrates the flow of practices (clusters) through the trial. Out of the 388 practices that were invited, 146 agreed to participate. One practice withdrew after randomization, before the outreach visit. The practices and patients in the intervention and control groups were similar with regards to baseline characteristics (Table 1).

**Table 1:**
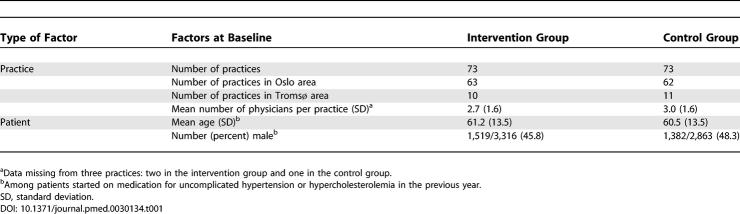
Baseline Characteristics

### Data Collection

**Figure 2:**
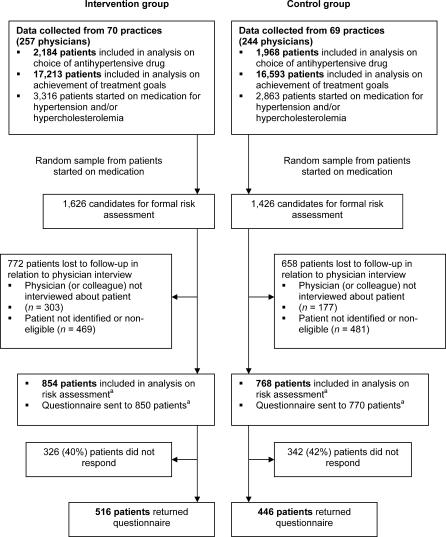
Patient Populations in Analyses ^a^Questionnaires were not sent to some patients included in the analysis of risk assessment (patient had died, unknown address, etc.), and some patients were excluded from the analysis of risk assessment after questionnaires had been sent to them.

For seven of the 146 participating practices we were unable to collect medical record data for various reasons (Figure 1). One of the remaining 139 practices was not included in the analyses involving estimation of cardiovascular risk (three secondary outcomes) because of an error during data collection. The numbers of patients included in the analyses are shown in Figure 2.

We interviewed 339 physicians about their use of risk assessment tools. For another 108 physicians, information was collected via a colleague at the practice, covering a total of 89% of all physicians.

For the questionnaire, which was sent to 1,620 patients, we achieved a response rate of 59%.

### Primary Outcomes

The main findings are presented in Table 2. Thiazide prescribing increased from 5.8% to 17.3% in the experimental group, and from 8.8% to 11.1% in the control group. Thus, prescribing of thiazides was significantly higher in the experimental group (relative risk 1.94; 95% confidence interval [CI]1.49–2.49). Little or no effect was detected for the two other main outcomes.

**Table 2:**
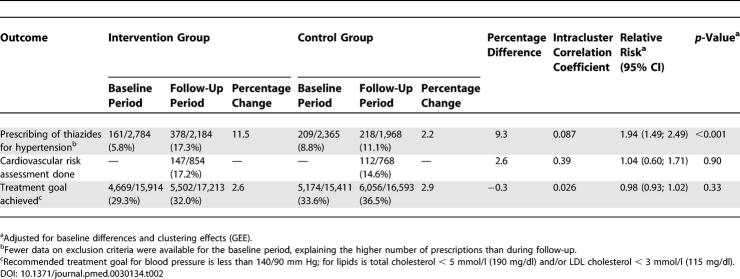
Primary Outcomes

### Secondary Outcomes

The results for the secondary outcomes are reported in Table 3. There were no statistically significant differences for these outcomes, except for those related to choice of antihypertensive drug. The distribution of responses to the patient questionnaire was similar in the two groups.

**Table 3:**
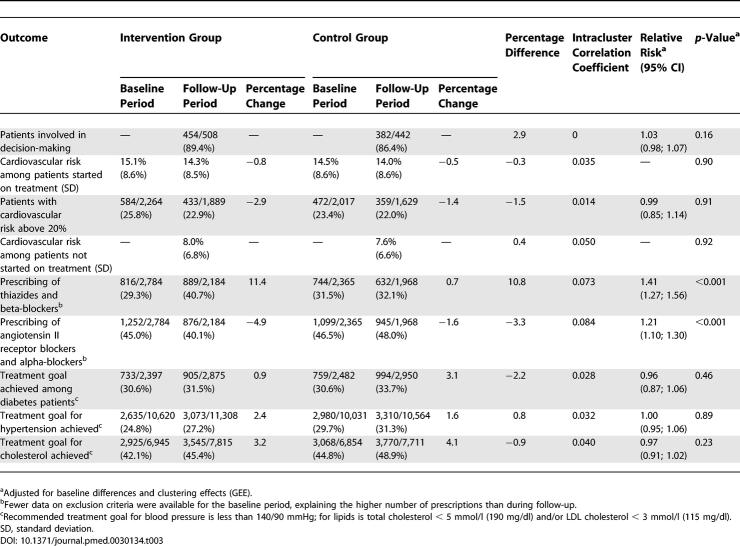
Secondary Outcomes

## Discussion

The intervention had an impact on prescribing patterns, but for other outcomes no statistically significant effects were found.

### Strengths and Weaknesses of the Study

Compared to other evaluations of quality improvement interventions, the size of the study and use of rigorous scientific methods are the main strengths of our study. The risk of selection bias was minimized by the randomized design. The impact of random errors was minimized by including 146 practices with 500 physicians and a large number of patients (between 950 and 33,800 per outcome).

Several assumptions had to be made during data analysis, such as assuming that patients were not on medication if no prior prescription for antihypertensive therapy was recorded. Some of the patients proved to be on medication that had been prescribed elsewhere. Similarly, we relied on routinely collected clinical data for coding patients as smokers or nonsmokers, and these data may be incomplete even though we searched the free-text notes area of the medical records for information on smoking. However, we can assume that these errors are evenly distributed between the intervention and control groups, and thus should not introduce biases into the analyses.

One outcome was based on self-report by physicians, and another on self-report by patients. It is possible that physicians in the experimental group tended to report a higher use of risk assessment tools since this had been promoted to them. However, the reported use of risk assessment tools was similar in the two groups.

Practices in only two areas of Norway were invited to participate in the trial, and only 38% of them were included. Some practices were not invited because they did not use a medical record system compatible with our software. Thus, the practices in the trial are not necessarily representative for all general practices in Norway. Compared to national statistics, the proportion of practices with only one or two physicians was higher in our study (48% versus a national average of 30%).

### Study Findings in Relation to the Overall Evidence

Walsh and colleagues recently conducted a systematic review of studies evaluating quality improvement interventions for the management of hypertension [23]. They included four randomized controlled trials of interventions targeting the choice of antihypertensive drug [24–27]. None of these trials reported statistically significant results. The point estimates for effect sizes ranged from −2.6% to 6%. The corresponding figure for our study is 9.3%. The review also included some nonrandomized studies, and the authors' overall conclusion was that “the median increase in percentage of providers adhering to recommendations was 3.0%” (interquartile range 1.0%–5.5%).

One randomized trial published too recently to be included in the systematic review was also aimed at increasing the prescribing of thiazides [28]. The intervention involved case-based educational modules and personal prescribing feedback. The investigators reported an absolute effect size slightly better than ours: an increase in thiazide prescribing of 11.5% (95% CI 4.0%–18.8%). Thus, although the results from trials targeting professional behavior have often been disappointing, there are some encouraging findings, including those reported by us.

Walsh et al. also reviewed trials of interventions aimed at improving the achievement of treatment goals for patients on antihypertensive therapy. They found a median increase in the proportion of patients achieving recommended targets of systolic and diastolic blood pressure of 16.2% (interquartile range 10.3%–32.2%) and 6.0% (interquartile range 1.5%–17.5%), respectively. Nonrandomized trials were also included in this analysis. The greatest effect sizes were achieved with interventions involving organizational change and patient education. Our intervention included no such components, apart from giving physicians the ability to print out patient education materials incorporated into their medical record system.

### Possible Explanations

Some specific areas of professional behavior may be more difficult to influence than others. For instance, physicians are under immense pressure from the pharmaceutical industry to choose antihypertensive drugs other than thiazides, which are off-patent drugs available at a low price. On the other hand, achieving treatment goals and conducting cardiovascular risk assessment are uncontroversial recommendations that are encouraged by practically all stakeholders. Attitudes and lack of knowledge among physicians are likely to be the main barriers to adhering to these recommendations, and our intervention was specifically designed to address these [19].

Despite our targeted intervention, we did not succeed in having an impact on these behaviors. One explanation may be that achieving treatment goals is a patient outcome, which depends on compliance of patients as well as physicians. Our intervention was mainly directed at physician behavior. We have no good explanation for the lack of effect on the use of risk assessment tools. A process evaluation is underway, to identify why the intervention did not have an effect on various outcomes across practices.

The outreach visits were conducted as group sessions with all the physicians in the practice. It is likely that visiting physicians individually is more effective [29], but this is also more costly to implement. Our intervention was multifaceted, and it is not possible to say which of the various components were the most important for the overall effectiveness. The planned process evaluation may provide some insight.

Although we managed to nearly double the prescribing of thiazides, it can be argued that the effect is far from satisfactory. The use of thiazides is still very limited in Norway, and although we achieved a rate of 17% for the prescribing of thiazides, this is low relative to other countries and relative to what might be considered appropriate [30].

An alternative strategy for quality improvement is the use of regulatory measures to enforce treatment protocols. Legislation went into effect in March 2004 that requires Norwegian doctors to prescribe thiazides as the first-choice antihypertensive drug if the patients are to have their drug costs reimbursed through the national health insurance system. However, the effect of such legislative measures is not well documented. Our results are in line with those of other randomized trials that have found outreach visits to be effective at changing prescribing, with similar effect sizes (median adjusted risk difference 3%, range 0%–7.5%, for 12 studies), while effects on other outcomes are much less consistent [31]. We did not find our intervention to be effective for other behaviors. We report on the cost effectiveness of our intervention in a companion article [32].

## Supporting Information

### Trial Registration

This trial has the registration number ISRCTN48751230 in the International Standard Randomized Controlled Trial Number Register.

Found at: http://www.controlled-trials.com/isrctn/trial/|/0/48751230.html.

Protocol S1 Trial Protocol (310 KB PDF)

**Box 1. Components of the Tailored Intervention**

**Educational Outreach Visit**

• Presentation focusing on three main messages: (1) relevance of risk estimation and how to do it, including strategies on how to communicate information about risk to patients, (2) evidence of the effectiveness of thiazides and that fears of adverse effects are unjustified, and pointing out the consensus that exists among guidelines (attention also directed to the importance of clinically relevant endpoints when studies are quoted), and (3) clear recommendations justified by referring to the high degree of consensus among guidelines

• Guidelines handed out, directing attention to the authors (opinion leaders)

Audit and Feedback at Outreach Visit

• To what extent treatment goals are currently being achieved

• Drug choice profile on antihypertensives

• Level of risk among patients on treatment, compared to a sample (men, 40–65 y) not on treatment

Computerized Reminders

• Risk assessment

• First-choice antihypertensive drugs

• Treatment goals

Risk Assessment Tools (Software and Charts)

Patient Information Material

• The relationship between single risk factors and global risk

• Thiazides and beta-blockers.

• Treatment goals

Follow-Up

• Telephone call within 1–3 d to check that software had not led to any difficulties

• Short telephone interview with each physician after 1–3 mo

## Abbreviations

CI: confidence interval
GEE: generalized estimating equations
LDL: low-density lipoprotein
