# Supplementary material for: Rational Prescribing in Primary Care (RaPP): A Cluster Randomized Trial of a Tailored Intervention
Source: PLoS Med. 2006 Jun 6;3(6):e134. doi: 10.1371/journal.pmed.0030134 (PMC1472695; doi:10.1371/journal.pmed.0030134)
Supplement: Supplementary file 1 [file pmed.0030134.sd001.pdf]

BMC Health Services Research

BioMed Central

Study protocol

Open Access

Rational Prescribing in Primary Care (RaPP-trial). A randomised trial of a tailored intervention to improve prescribing of antihypertensive and cholesterol-lowering drugs in general practice [ISRCTN48751230]

Atle Fretheim*[1], Andrew D Oxman[1], Shaun Treweek[1] and Arild Bjørndal[2]

Address: [1]Department of Health Services Research, Norwegian Directorate for Health and Social Affairs, N-0031 Oslo, Norway and [2]Division of Knowledge Management, Norwegian Directorate for Health and Social Affairs, N-0031 Oslo, Norway

Email: Atle Fretheim* - atle.fretheim@shdir.no; Andrew D Oxman - oxman@online.no; Shaun Treweek - shaun.treweek@shdir.no; Arild Bjørndal - arild.bjorndal@samfunnsmed.uio.no

* Corresponding author

Published: 27 February 2003

BMC Health Services Research 2003, **3**:5

This article is available from: http://www.biomedcentral.com/1472-6963/3/5

Received: 7 December 2002
Accepted: 27 February 2003

© 2003 Fretheim et al; licensee BioMed Central Ltd. This is an Open Access article: verbatim copying and redistribution of this article are permitted in all media for any purpose, provided this notice is preserved along with the article's original URL.

Abstract

Background: The underlying reasons for differences between clinical practice and systematically developed guidelines vary from one clinical problem to another. It is therefore logical to tailor strategies to support the implementation of guidelines to address identified barriers to change. The objective of this trial is to evaluate the effects of a tailored intervention to support the implementation of systematically developed guidelines for the use of antihypertensive and cholesterol-lowering drugs for the primary prevention of cardiovascular disease.

Methods/Design: Unblinded, cluster-randomised trial. 150 general practices will be recruited from two geographical areas in Norway, and randomised to the intervention or control group (passive dissemination of guidelines). Outcomes will be measured for all eligible patients seen in the participating practices during one year after the intervention. A multifaceted intervention has been tailored to address identified barriers to change. Key components are an educational outreach visit with audit and feedback, and computerised reminders. Pharmacists will conduct the visits. During the outreach visit the main recommendations will be presented and software will be installed that links to the electronic medical record systems used in the participating practices. The software will perform an audit that will be fed back during the visit, present pop-up reminders for patients with high blood pressure or cholesterol, and provide a cardiovascular risk calculator and patient education material. The main outcomes are the proportions of 1) first time prescriptions for hypertension where thiazides are not prescribed, 2) patients not assessed for cardiovascular risk before prescribing antihypertensive or cholesterol-lowering drugs, and 3) patients treated for hypertension or high cholesterol for three months or more who have not achieved recommended treatment goals.

Background

Hypertension and hypercholesterolaemia are common problems in general practice and have important consequences for patients and the use of healthcare resources. In Norway the cost of prescription drugs for hypertension and hypercholesterolaemia accounts for 18% of the total public expenditure on drugs [1]. Cardiovascular disease is

a common cause of death and an important cause of morbidity in Norway.

Appropriate use of antihypertensive and cholesterol-lowering drugs for the primary prevention of cardiovascular disease can have important benefits, whereas inappropriate prescribing entails substantial costs, as well as undesirable consequences for patients.

There is high quality evidence from well-designed randomised trials of the effects of antihypertensive and cholesterol-lowering drugs for the primary prevention of cardiovascular disease [2,3]. There are also a number of clinical practice guidelines, often with conflicting recommendations, e.g on the choice of drug or threshold to start treatment [4]. Based on a review of previous guidelines and the underlying evidence, we developed guidelines for prescribing anthihypertensive and cholesterol-lowering drugs for the primary prevention of cardiovascular disease in Norwegian general practice [5–7].

There is potential for improvement for three key recommendations: cardiovascular risk is commonly not measured before starting treatment [8], thiazides are underutilised when initiating treatment for hypertension [1], and treatment goals are frequently not achieved for both blood pressure and cholesterol [9,10]. We have therefore developed an intervention aimed at supporting the implementation of these three recommendations and planned a trial to assess the effectiveness and costs of this intervention.

The results of evaluations of interventions to improve clinical practice vary and large effects are not common [11,12]. As with any behaviour, professional behaviour is difficult to change. Several strategies have been used. Some of them, like passive dissemination of clinical practice guidelines, have little or no effect on practice [12,13]. Other, more active strategies, like educational outreach visits [14] and multifaceted interventions [11], are more effective, but require more resources [11]. The underlying reasons for differences between clinical practice and systematically developed guidelines vary from one clinical problem to another and from one clinician to another [15,16]. It is therefore logical to tailor strategies to support the implementation of guidelines to address identified barriers to change [15]. However, the effects of such interventions remain uncertain [16,17]. They should be rigorously evaluated before they are widely used. Because the effects are expected to be moderate, at best, and biases, particularly non-equivalent comparison groups, can easily distort the results of evaluations, cluster-randomised trials (in which healthcare professionals or groups of professionals, rather than patients are randomised) are more likely to provide valid results than other research designs, such as controlled before-after or time-series studies [18].

Objectives
The primary objective of this trial is to evaluate the effects of a tailored intervention to support the implementation of guidelines by Norwegian general practitioners for the use of antihypertensive and cholesterol-lowering drugs for the primary prevention of cardiovascular disease. Our hypothesis is that the tailored intervention will be more effective than passive dissemination of the guidelines at reducing the proportions of first time prescriptions for hypertension where thiazides are not prescribed, patients not assessed for cardiovascular risk before prescribing antihypertensive or cholesterol-lowering drugs, and patients treated for hypertension or high cholesterol for three months or more who have not achieved recommended treatment goals.

Secondary objectives are to estimate the use of resources associated with the intervention and the effects of the intervention on other relevant outcomes. We anticipate that passive dissemination of guidelines, which is unavoidable for published guidelines, will have little effect on the outcomes of interest [11–13] and is therefore an appropriate control-intervention.

A process evaluation, to understand the effects or lack of effects of the intervention, and an economic analysis, if the intervention is effective, are described in separate protocols.

Methods
Design
The main hypotheses will be tested using a cluster-randomised controlled trial comparing outcomes between the intervention and control groups at follow up. General practices will be randomised to receive a tailored intervention to support implementing the guidelines or to a control group (see Figure 1). Data will be collected for all eligible patients from participating practices for one year after the outreach visit. For the control group data will be collected for one year after publication of the guidelines in the Journal of the Norwegian Medical Association.

Participants
All general practices in or near to Oslo or Tromsø in Norway that use one of two electronic medical record systems (WinMed or ProfDoc Vision) are eligible for the trial. Over 80% of Norwegian general practices use one of three electronic medical record systems, and these are used routinely during consultations. The two restrictions are pragmatic. 1) The computer software that will be used does not yet function with the third electronic medical record system. 2) We have recruited four pharmacists to carry out

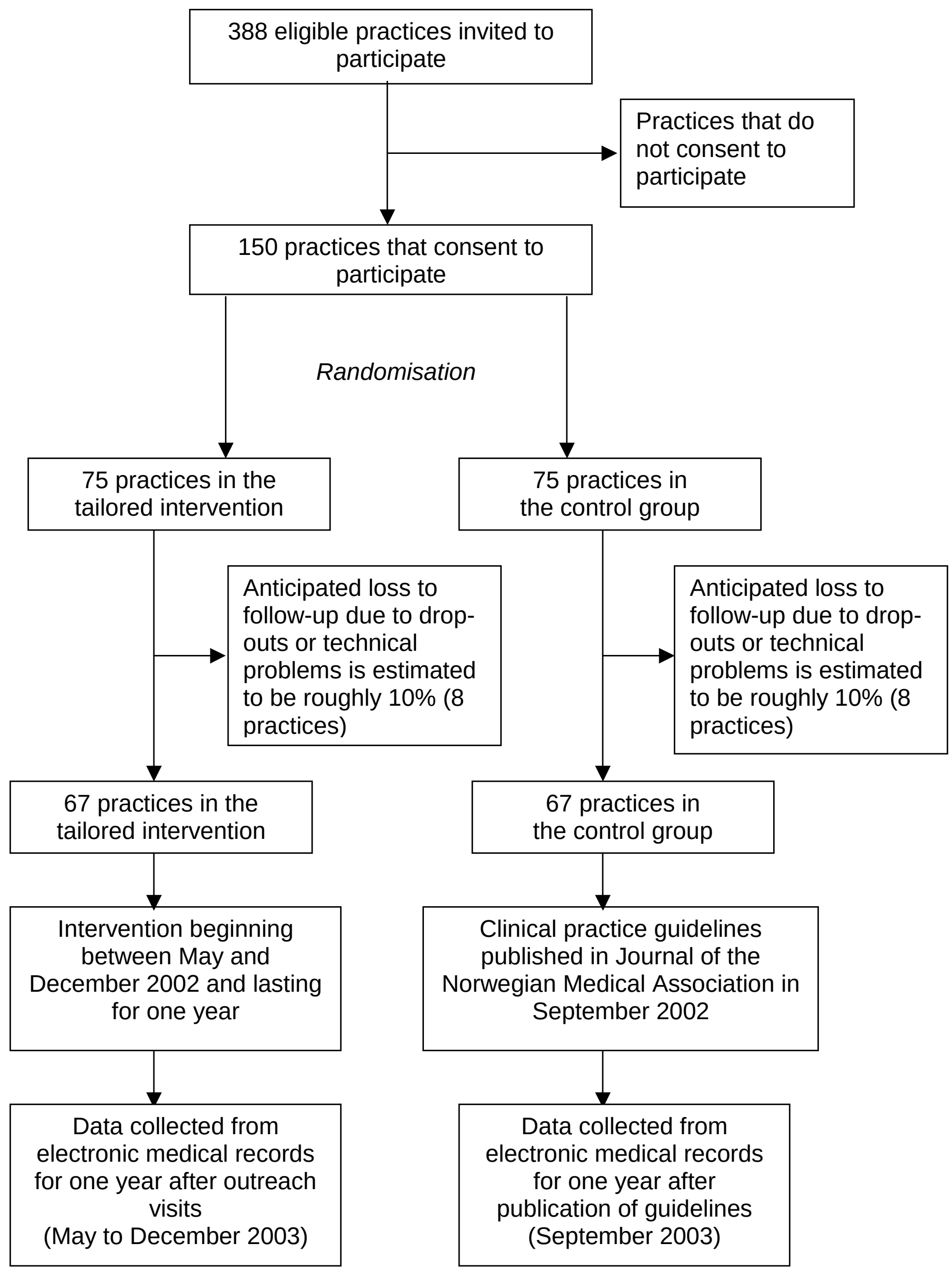

Figure 1
Flow of practices through trial

Table 1: Elements of the tailored intervention

**Educational outreach visit**
• Presentation focusing on three main messages:
1. Relevance of risk estimation and how to do it, including strategies on how to communicate information about risk to patients.
2. Information on evidence in support of effect and the unjustified fear of adverse effects regarding thiazides, pointing at the consensus that exists among guidelines. Attention also directed to the importance of clinically relevant endpoints when studies are quoted.
3. Clear recommendations justified by referring to high degree of consensus among guidelines.
• Guidelines handed out, directing attention to the authors (opinion leaders)
**Audit & feed-back at outreach visit**
• To what extent treatment goals are achieved.
• Drug-choice profile on antihypertensives
• Level of risk among patients on treatment, compared to a sample (men 40–65 years) not on treatment
**Computerised reminders**
• Risk assessment
• First-choice antihypertensive drugs
• Treatment goals
**Risk assessment tools as charts and in electronic format**
**Patient-information material**
• The relationship between single risk factors and global risk
• Thiazides and beta-blockers.
• Treatment goals
**Follow-up**
• Telephone call to the practice within 1–3 days to check that software has not led to any difficulties
• Short telephone call to each physician after 1–3 months

the outreach visits. Three of them live in the Oslo area and one near to Tromsø. We have chosen to recruit practices in the corresponding geographical areas. There are 388 general practices that meet these inclusion criteria. All of these practices will be invited to participate.

Intervention

We developed our intervention through a process of identifying barriers to implementation of the recommendations and subsequently coming up with suggestions of measures that may facilitate change by specifically addressing the barriers ("tailoring"). This was mainly done through active reflection and discussions in a group of three physicians with experience from general practice (including two of the authors, AF and ADO). In order to structure the reflection we used a worksheet designed to facilitate the discussion by listing possible factors that may act as barriers within the practice environment, within the professional environment, and related to physicians' knowledge, skills and attitudes [19]. We also took into account information gathered from a survey of general practitioners, experience from our own research, and from discussion with physicians during pilot testing of the intervention.

In order to identify previously executed trials of interventions targeted at the management of hypertension and/or elevated cholesterol in general practice, the trial register of the Cochrane Group of Effective Practice and Organisation of Care (EPOC) was searched in July 2001 (See Additional File 1: Appendix) [20].

Table 1 describes the various elements of our intervention.

The practical implementation of the intervention will be initiated through an educational outreach visit carried out by pharmacists recruited specifically for this purpose. No specific qualifications will be demanded, and the pharmacists will be given the necessary training during two weeks in our department. This training will cover basic research methods, the content of the clinical practice guidelines, and two days dedicated to communication techniques.

During the outreach visit the main elements of the guideline will be presented, with special emphasis on risk estimation, choice of first-line drugs, and treatment goals. A printed copy of the guideline and a one-page version will be given to the physicians, including a chart to aid the estimation of cardiovascular risk.

During the visit a software package will be installed. This enables us to extract data and immediately, during the visit, present the physicians with data on their performance concerning risk estimation, choice of antihypertensive drugs and achievement of treatment goals ("audit and

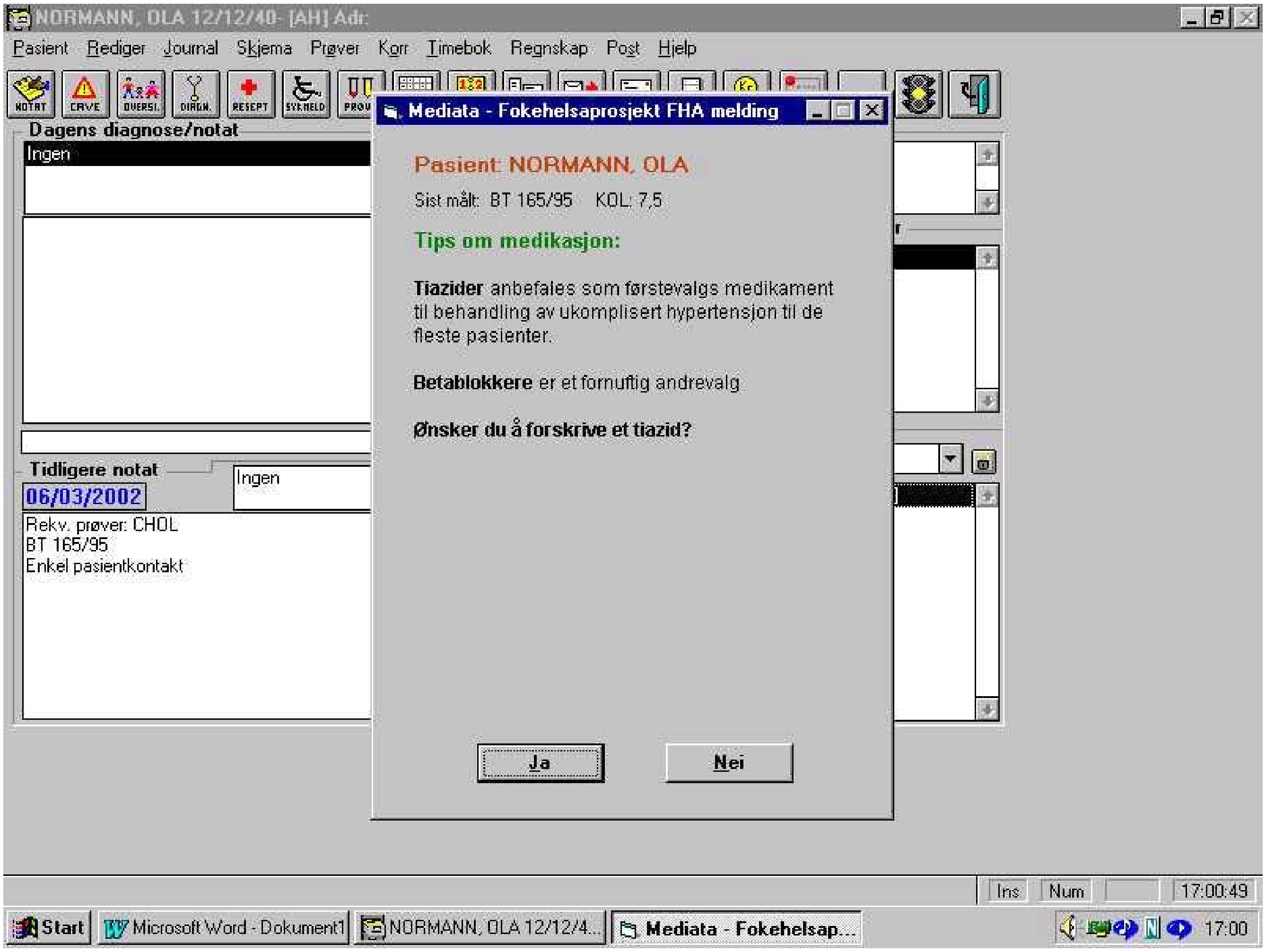

Figure 2

Example of pop-up on physician's computer screen. The pop-ups provide various reminders to the physician related to specific patients. For example, if the most recent blood pressure recorded within the last six months is above 140/90 mm Hg, the physician will be asked if antihypertensive therapy is being considered when the patient's medical record is opened. If the physician responds "Yes" the pop-up in the figure appears, which reminds the physician that thiazides are first-choice drug for most cases of uncomplicated hypertension and asks if he/she would like to prescribe a thiazide. If the response is "Yes" the next pop-up provides brand names of available thiazide-drugs.

feed-back"). The software package also includes computerized reminders using "pop-ups" on the computer screen (see Figure 2). These are triggered at the patient's first visit following a recording of an elevated blood pressure- or cholesterol-level (blood pressure > 140/90 mm Hg or total-cholesterol > 5 mmol/L or LDL-cholesterol > 3 mmol/L). The physician can easily close the "pop-ups" each time they appear. We will not record how often this is done.

If the patient has not been prescribed blood-pressure- or cholesterol-lowering drugs the physician is reminded of the recommendation to carry out cardiovascular risk assessment and is offered to start a computerized risk assessment tool (SmartHeart). Recommendations on choice of drugs are also given and the physician is also given the option of printing out patient information material.

If the patient has been prescribed blood-pressure- or cholesterol-lowering drugs, the "pop-up" will remind the physician of recommended treatment goals and ask if the physician would like to print out patient information material.

At the outreach visit a follow-up phone-call with each individual physician will be scheduled for 1–3 months after the initial visit and will focus on what questions, problems or concerns the physicians may have.

The pharmacists will complete a questionnaire after each outreach visit, enquiring about technical problems, physician-attitudes, and the pharmacist's own impression of the session.

One to two days after the visit a member of the research team will call the office to confirm that they are not experiencing problems as a result of our visit.

Outcome measures

We have chosen three main outcome measures, all aimed at physician-behaviour regarding the pharmacological management of primary prevention of cardiovascular disease for the 12 months following the outreach visit. Patients with a recorded cardiovascular diagnosis are excluded, with the exception of the third main outcome. Because antihypertensive drugs are also being prescribed for the treatment of thyrotoxicosis and migraine, we have excluded data from patients where these diagnoses are recorded. Patients are considered as previously untreated if they have hypertension (blood pressure > 140/90 mmHg) and/or hypercholesterolaemia (total-cholesterol > 5 mmol/L or LDL-cholesterol > 3 mmol/L) and no prescription for the corresponding medication has been recorded for 24 months preceding the outreach visit.

Main outcomes

• The proportion of prescriptions of other antihypertensives than thiazides to patients who are being prescribed antihypertensive drugs for the first time. (Measurement: data drawn from the practice medical record system.)

• The proportion of patients where the level of cardiovascular risk has not been estimated among all those started on antihypertensive or cholesterol lowering treatment. (Method: Physicians are given the names of the patients they have put on treatment and are asked if the cardiovascular risk has been evaluated, and if so: what the level was.)

• Proportion of patients with a recorded level of cholesterol (total or LDL) or blood pressure not satisfying the specified treatment goals, among all patients on the corresponding treatment for at least three months. For cholesterol we have decided to also include patients on secondary prevention therapy since the treatment goals are similar. (Measurement: data drawn from the practice medical record system.)

We will also investigate the following outcomes

• The proportion of patients reporting that they were not involved in the decision-making process before drug treatment for hypertension and/or elevated cholesterol was started. (Measurement: questionnaire sent to patients by their physician, and returned anonymously to the research team.)

• Level of risk among patients started on treatment. (Measurement: Data drawn from the practice medical record system, and entered into a version of the Framingham-equation [21].)

• Proportion of patients with risk under 20% receiving treatment among those receiving treatment. (Measurement: Data drawn for the practice medical record system, and entered into a version of the Framingham-equation.)

• Level of risk among patients not started on treatment where blood pressure and cholesterol level has been recorded. (Measurement: Data on drawn from the practice medical record system, and entered into a version of the Framingham-equation.)

• Proportion of prescriptions of other antihypertensives than thiazides and beta-blockers to patients who are being prescribed antihypertensive drugs for the first time. (Measurement: data drawn from the practice medical record system.)

• Proportion of angiotensin-II-receptor blockers and/or alpha-blockers among prescripions of antihypertensives to patients who are being prescribed antihypertensive drugs for the first time. (Measurement: data drawn from the practice medical record system.)

• Subgroup-analysis for patients with diabetes: Proportion of patients with a recorded level of cholesterol (total or LDL) or hypertension not satisfying the specified treatment goals, among all patients on the corresponding treatment. For cholesterol we have decided to also include patients on secondary prevention therapy since the treatment goals are similar. (Measurement: data drawn from the practice medical record system.)

• Proportion of patients not reaching the specified treatment goal for blood pressure (Measurement: data drawn from the practice medical record system.)

• Proportion of patients not reaching the specified treatment goal for cholesterol (Measurement: data drawn from the practice medical record system.)

Economic analysis

We will record parameters that describe costs related to our intervention:

• Salary-costs for pharmacists

• Time spent arranging for appointments at the doctors' practices

• Travel expenses in connection with each outreach visit

• Amount of physician-time spent at each visit

• Materials

• Training

• Other direct expenses related to the implementation of the intervention

The economic analysis, which will only be conducted if the intervention is effective, will be described in a separate protocol.

Pilot study

The intervention has been tested on a small group of general practitioners. The first 10 practices that receive the intervention will be excluded from the study and defined as a pilot-group if major practical problems arise.

Recruitment and randomisation

Block-randomisation was done within each geographical stratum: the Oslo-area and Northern Norway. The size of the blocks varied randomly between 2, 4 and 6. A colleague not directly involved in our research project generated the allocation-list using software from http://www.randomisation.com. We gave her ID-numbers representing each recruited practice, and she informed us whether the practice was allocated to the intervention- or control-group.

Baseline data

Baseline data (12 months before the intervention) will be collected for all outcomes on prescribing and measurements of blood pressure or cholesterol.

Ethics

The doctors who are invited to participate in the study will be given information about the objectives of the study and the practical impact it may have on their practice. They will be told that they will be randomised to an experimental or control group. We will obtain written consent from all practices. The aim of this study is to improve quality of care and thus should not imply any risk for the patients affected by the study. It is difficult to imagine that our intervention could worsen the quality of care. We have presented the project for the Regional Committee of Research Ethics and they did not consider it necessary to submit the protocol for approval.

The Norwegian Data Inspectorate has approved the handling of data. In accordance with regulations we have assessed the risk of misuse of the data we will be collecting. The risk is minimal due to the fact that the data are linked to a reference number that can only be linked to the specific patient by accessing the computer-system at the practice of the patient's physician using a program developed for this task.

Sample size and statistics

In order to demonstrate a 25% relative reduction, with a power of 80% and a statistical significance level of 5%, in outcome measures between the control and intervention groups, we estimated a need for a sample of almost 140 practices in total (Cluster Randomisation Sample Size Calculator ver 1.0.2, Health Services Research Unit, Aberdeen University). We assumed that none of the main outcomes would be less than 50% in the control group and that the average number of patients included per practice would be around 10 per outcome measure (three patients per physician, and three physicians per practice). This was based on sales figures of anti-hypertensive drugs [1], a survey on the usage of risk assessment tools [8], published figures on achievement of treatment goals [9,10], estimates on the prevalence of hypertension-treatment (9% of those aged over 25) [22], and data we previously had collected from a group of practices. The sample size also takes into account the need to adjust for intracluster correlation, which is a consequence of randomising at one level (clinical practices) and analysing at another (patients). The adjusting factor was conservatively estimated to be 0.2, based on data from a previous study [17].

We will carry out post-test comparisons between the intervention- and control-group using cluster-adjusted chi-square and T-tests. Analysis of covariance may be used to adjust for imbalance of baseline levels between the intervention- and control-groups [23].

Competing interests

All the authors are employed by the Norwegian government, which has a substantial interest in improving professional practice in Norway and in containing the costs of healthcare. The clinical practice guidelines and the study design have not been endorsed by the government or any other organisation and represent the views of the authors and not necessarily those of the government. No other competing interests declared.

Authors' contributions

ADO and AB had the original idea for the study. AF prepared the draft of the study protocol in cooperation with ADO. ST and AB provided important input during the planning of the study. All authors have approved the final manuscript.

Additional material

Additional File 1

1. Appendix.doc (Search in the trial register of the Cochrane Group of Effective Practice and Organisation of Care [EPOC])
Click here for file
[http://www.biomedcentral.com/content/supplementary/1472-6963-3-5-S1.doc]

Acknowledgements

The Norwegian Ministry of Health funds the study. Pfizer provided SmartHeart, a computer program for calculating cardiovascular risk, without conditions other than being mentioned under acknowledgements. Logos and information relating the software to Pfizer were removed, and we do not consider there to be any significant conflict between their interests and the possible results of our study. Mediata prepared the software package used to audit practices, provide reminders and patient information, and collect data. Signe Flottorp, Kari Håvelsrud and Morten Aaserud have provided helpful advice and support.

References

1. WHO Collaborating Centre for Drug Statistics Methodology **Drug Consumption in Norway 1996 - 2000** *Oslo (Edited by: Rønning M) Oslo* 2001,
2. Collins R, Peto R, MacMahon S, Hebert P, Fiebach NH, Eberlein KA, Godwin J, Qizilbash N, Taylor JO and Hennekens CH **Blood pressure, stroke, and coronary heart disease. Part 2, Short-term reductions in blood pressure: overview of randomised drug trials in their epidemiological context** *Lancet* 1990, **335:**827-838
3. LaRosa JC, He J and Vupputuri S **Effect of statins on risk of coronary disease: a meta-analysis of randomized controlled trials** *JAMA* 1999, **282:**2340-2346
4. Fretheim A, Williams JW, Oxman AD and Herrin J **The relation between methods and recommendations in clinical practice guidelines for hypertension and hyperlipidemia** *The Journal of Family Practice* 2002, **51:**963-968
5. Fretheim A, Bjørndal A, Oxman AD, Dyrdal A, Golding M, Ose L, Reikvam Å and Teisberg P **Guidelines for pharmacological primary prevention of cardiovascular disease: who should be treated?** *Tidsskr Nor Lægeforen* 2002, **122:**2277-2281
6. Fretheim A, Bjørndal A, Oxman AD, Dyrdal A, Golding M, Ose L, Reikvam Å and Teisberg P **Which antihypertensive drugs should be used in the primary prevention of cardiovascular disease?** *Tidsskr Nor Lægeforen* 2002, **122:**2283-2286
7. Fretheim A, Bjørndal A, Oxman AD, Dyrdal A, Golding M, Ose L, Reikvam Å and Teisberg P **Which cholesterol-lowering drugs should be used in the primary prevention of cardiovascular disease?** *Tidsskr Nor Lægeforen* 2002, **122:**2287-2288
8. Fretheim A, Håvelsrud K, Flottorp S and Oxman AD **Do fees for service and regulations for reimbursement influence practice?** *Tidsskr Nor Lægeforen* 2003,
9. Svilaas A, Risberg K, Thoresen M and Ose L **Lipid treatment goals achieved in patients treated with statin drugs in Norwegian general practice** *Am J Cardiol* 2000, **86:**1250-3, A6
10. Westheim A, Klemetsrud T, Tretli S, Stokke HP and Olsen H **Blood pressure levels in treated hypertensive patients in general practice in Norway** *Blood Press* 2001, **10:**37-42
11. Grimshaw JM, Shirran L, Thomas R, Mowatt G, Fraser C, Bero L, Grilli R, Harvey E, Oxman A and O'Brien MA **Changing provider behavior: an overview of systematic reviews of interventions** *Med Care* 2001, **39:**II2-45
12. Oxman AD, Thomson MA, Davis DA and Haynes RB **No magic bullets: a systematic review of 102 trials of interventions to improve professional practice** *CMAJ* 1995, **153:**1423-1431
13. Freemantle N, Harvey EL, Wolf F, Grimshaw JM, Grilli R and Bero LA **Printed educational materials: effects on professional practice and health care outcomes** *In: The Cochrane Library, Issue 4, Oxford: Update Software* 2001,
14. Thomson O'Brien MA, Oxman AD, Davis DA, Haynes RB, Freemantle N and Harvey EL **Educational outreach visits: effects on professional practice and health care outcomes** *In: The Cochrane Library, Issue 4, Oxford: Update Software* 2001,
15. Oxman AD and Flottorp S **An overview of strategies to promote implementation of evidence base health care** *Evidence Based Practice (Edited by: Silagy C and Haines A) London, BMJ Books* 2001, **2nd:**101-119
16. Flottorp S, Håvelsrud K and Oxman AD **Evaluation of a pragmatic trial of interventions to implement guidelines in primary care - why is it so hard to change practice?** *Fam Pract* 2003,
17. Flottorp S, Oxman AD, Håvelsrud K, Treweek S and Herrin J **A cluster randomised trial of tailored interventions to improve the management of urinary tract infections and sore throat** *BMJ* 2002, **325:**367-370
18. Campbell MK, Steen N, Grimshaw JM, Eccles M, Mollison J and Lombard C **Design and statistical issues in implementation research** *Changing Professional Practice (Edited by: Thorsen T and Mäkelä M) Copenhagen, Danish Institute for Health Services Research and Development* 1999, 57-76[http://www.dsi.dk/projects/cpp/Monograph/DSI9905.pdf]
19. Flottorp S and Oxman AD **Tailoring interventions to improve the management of urinary tract infections and sore throat: a pragmatic study using qualitative methods** *BMC Health Services Research* 2003, **3:**3
20. **EPOC specialised register** *http://www.epoc.uottawa.ca/register.htm* 2001,
21. Anderson KM, Wilson PW, Odell PM and Kannel WB **An updated coronary risk profile. A statement for health professionals** *Circulation* 1991, **83:**356-362
22. Komiteen for medisinsk teknologivurdering **Behandling av mild hypertensjon** *Oslo* 1997, [http://www.forskningsradet.no/bibliotek/publikasjoner/hypertensjon_r12_norsk]
23. Vickers AJ and Altman DG **Analysing controlled trials with baseline and follow up measurements** *BMJ* 2001, **323:**1123-1124

Pre-publication history

The pre-publication history for this paper can be accessed here:

http://www.biomedcentral.com/1472-6963/3/5/prepub

Publish with **BioMed Central** and every scientist can read your work free of charge

"BioMed Central will be the most significant development for disseminating the results of biomedical research in our lifetime."
Sir Paul Nurse, Cancer Research UK

Your research papers will be:

- available free of charge to the entire biomedical community
- peer reviewed and published immediately upon acceptance
- cited in PubMed and archived on PubMed Central
- yours — you keep the copyright

Submit your manuscript here:
http://www.biomedcentral.com/info/publishing_adv.asp

BioMedcentral
